# Pre-trained Deep Convolution Neural Network Model With Attention for Speech Emotion Recognition

**DOI:** 10.3389/fphys.2021.643202

**Published:** 2021-03-02

**Authors:** Hua Zhang, Ruoyun Gou, Jili Shang, Fangyao Shen, Yifan Wu, Guojun Dai

**Affiliations:** ^1^School of Computer Science and Technology, HangZhou Dianzi University, Hangzhou, China; ^2^Key Laboratory of Network Multimedia Technology of Zhejiang Province, Zhejiang University, Hangzhou, China; ^3^Key Laboratory of Brain Machine Collaborative Intelligence of Zhejiang Province, HangzhouDianzi University, Hangzhou, China

**Keywords:** speech emotion recognition, deep convolutional neural network, attention mechanism, long short-term memory, deep neural network

## Abstract

Speech emotion recognition (SER) is a difficult and challenging task because of the affective variances between different speakers. The performances of SER are extremely reliant on the extracted features from speech signals. To establish an effective features extracting and classification model is still a challenging task. In this paper, we propose a new method for SER based on Deep Convolution Neural Network (DCNN) and Bidirectional Long Short-Term Memory with Attention (BLSTMwA) model (DCNN-BLSTMwA). We first preprocess the speech samples by data enhancement and datasets balancing. Secondly, we extract three-channel of log Mel-spectrograms (static, delta, and delta-delta) as DCNN input. Then the DCNN model pre-trained on ImageNet dataset is applied to generate the segment-level features. We stack these features of a sentence into utterance-level features. Next, we adopt BLSTM to learn the high-level emotional features for temporal summarization, followed by an attention layer which can focus on emotionally relevant features. Finally, the learned high-level emotional features are fed into the Deep Neural Network (DNN) to predict the final emotion. Experiments on EMO-DB and IEMOCAP database obtain the unweighted average recall (UAR) of 87.86 and 68.50%, respectively, which are better than most popular SER methods and demonstrate the effectiveness of our propose method.

## 1. Introduction

As the most natural and convenient medium in human communication, speech signals not only contain the linguistic information like semantic and language type, but also contain rich non-linguistic information, such as facial expression, speech emotion, and so on. In recent years, with the continuous development of artificial intelligence, speech emotion recognition (SER) plays a crucial role in human-machine interactions (Ayadi et al., 2011). More and more researchers are attracted by the study that computer automatically recognize speech emotions of people. Speech emotion recognition has become an attractive research topic in many fields, such as speaker's semantic and culture, but also contain a wealth of paralinguistic information, such as emotion.

Speech emotion recognition is under great challenges. Firstly, there are too few datasets in the speech field as it is difficult and time-consuming to build high-quality speech emotion database. Secondly, different data in the database has different speakers whose gender, age, language, and culture etc are different. Finally, the emotions in speech are often based on sentences rather than just certain words. So how to use LLDs and sentence-level features to improve the accuracy of emotion recognition is a difficult point in current research. The traditional speech emotion recognition methods usually contain three steps (Deng et al., 2014). The first step is data preprocessing, including data normalization, speech segmentation, and other operations. Next step is feature extraction from the speech signals using some machine learning algorithms. These features are usually called Low-Level Descriptors (LLDs), such as Fundamental Frequency(F0) (Origlia et al., 2010), Formant (Deng et al., 2013), Mel Frequency Cepstrum Coefficient (MFCC) (Milton et al., 2014), etc. Finally, appropriate classifiers are selected for speech emotion classification, including Support Vector Machine (SVM) (Chen et al., 2012), Gaussian Mixture Model (GMM) (Bhaykar et al., 2013), Hidden Markov model (Schuller et al., 2003), etc. However, a major disadvantage of those methods lies in the involved traditional machine learning technology which requires prior knowledge of all necessary features (such as fundamental frequency, energy, etc.) affecting emotion recognition. And the extraction process may lose some important information.

To address this problem, the deep learning techniques provide reasonable solutions in feature extraction for SER. One of the most popular deep learning methods is DNN, which have shown excellent performances in extracting discriminative features especially in image classification. For speech emotion recognition, using deep learning technology can automatically extract deep speech emotional features and learn the correlation between features. It has shown better performance compared with the traditional methods.

Originally, Han et al. (2014) proposed a DNN and ELM model in 2014, which adopted the highest energy fragments to train DNN model and extract effective emotional information. In 2014, Mao et al. (2014) first used convolutional neural network (CNN) to learn the emotional salient features of SER, and demonstrated the feasibility of CNN model on several benchmark data sets. Lee and Tashev (2015) used bidirectional long short-term memory (BLSTM, a special type of RNN) to extract high-level emotional representation which contained its temporal dynamics information. In 2016, Trigeorgis et al. (2016) proposed a convolutional RNN (CRNN) network which used the raw speech data to predict emotional changes.

Although DNN has achieved great success in SER, there are still some problems. First, the speech signal is quite different due to the variance of speaker's style, content, and environment. Second, DNN learned high-level feature representations from Low-Level Descriptors (LLDs) which cannot sufficiently extract emotional features. Then researchers began to use spectrograms to represent speech signals. The horizontal axis of spectrogram represents the information in time domain and the vertical axis represents the frequency information, making it a decent speech representation that retains the important emotional features of speech. Then CNN is used to automatically extract emotional features from spectrograms which has achieved superior performance in the field of SER.

In 2017, Badshah et al. (2017) used spectrograms and DCNN model to extract features related to speech emotion. They demonstrated the effectiveness of the method and achieved a good result of 84.3% on Berlin Emo-DB. Zhang et al. (2017) proposed a new method which directly to use three channels of log Mel-spectrograms as the pre-trained DCNN's input. Then, they used pyramid matching algorithm (DTPM) to normalize the segment-level features with unequal length. They verified the effectiveness of pre-trained DCNN model with 3-D log Mels on four speech databases. In 2018, Zheng et al. (2018) proposed a new SER model combine with convolutional neural network (CNN) and random forest (RF). They adopted CNN to extract the emotional features from spectrograms, and then used RF for classification. The satisfactory results proved that their model was robust and reasonable.

While spectrogram can retain emotional features well, there is an important and common problem in the above researches that the emotion labels of segments after speech segmentation are marked at the utterance-level. However, not all segments in an utterance contain emotional feature, such as silent frames and emotion irrelevant frames. Therefore, it is important to reduce the influence of these irrelevant segments. Attention mechanism can increase relatively high weights to emotion-related features, emphasizing the importance of these features, and reduce the influence of irrelevant features. It can help the network automatically focus on the emotion relevant segments and obtain discriminative features with utterance-level for SER.

Attention mechanism is adapted for speech emotion recognition work well (Mirsamadi et al., 2017). Zhao et al. (2018) proposed a new method combining Fully Convolutional Networks (FCNs) and attention-based RNNs for speech emotion recognition. The experimental results showed the high performance of the proposed method in IEMOCAP (Busso et al., 2008) and CHEAVD (Li et al., 2017) dataset. Mu et al. (2017) used distributed convolutional neural network (CNN) to automatically learn the emotion features from the raw speech spectrum, and they used bidirectional BRNN to obtain the time information from the CNN output. Finally, the output sequence of BRNN was weighted by attention mechanism algorithm to focus on the useful part of emotion. The weighted accuracy (WA) and unweighted accuracy (UA) of 64.08 and 56.41% were obtained from the IEMOCAP dataset, respectively. Lee et al. (2018) proposed a model combining the convolutional neural network with the attention mechanism and the text data. The promising experimental result in the CMU-MOSEI database proved the effectiveness of the combination of the two modalities.

**Figure 1:**
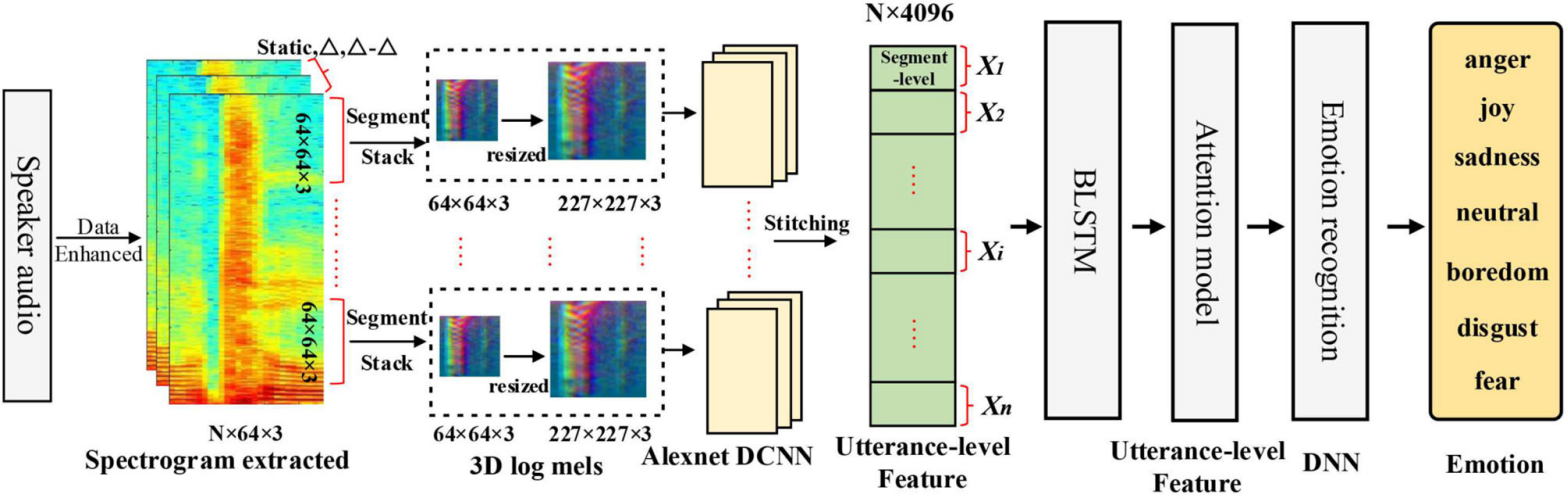
The overview of our proposed method using ADCNN-BLSTM model for SER.

Inspired by Zhang et al. (2017) and Zhao et al. (2018), in this paper, we propose a novel method based on DCNN and Bidirectional Long Short-Term Memory with attention model (DCNN-BLSTMwA). As illustrated in Figure 1, we first conduct data enhancement operation by adjusting different speech playing speed on the original speech data and use balancing datasets weight method to solve the problem of unbalanced emotion data distribution. Secondly, log Mel-Spectrograms (static, delta, delta-delta) of three channels are extracted as the DCNN input. And we initialize parameters by using pre-trained model on ImageNet dataset. Then, we fine-tune the DCNN with our speech data to extract segment-level features and all the segment-level features of a sentence are combined into an utterance-level feature as the input of BLSTM-Attention model. Next, the BLSTM further captures time-frequency relationship of utterance-level features, and the attention model is used to make the emotion features more prominent. After attention layer, we have extracted high-level utterance-level features. Finally, we adopt fully-connected DNN classifier for emotion classification. Abundant experiments on the Berlin Emotional database (EMO-DB) and the Interactive Emotional Dyadic Motion Capture database (IEMOCAP) demonstrate the stable and robust performance of our propose method. The main contributions of our paper can be summarized as follows:

1. To solve the problem of the small number of training samples for DCNN network training, we use data enhancement and speech segmentation to expand the number of samples. Firstly, we propose a data enhancement method based on overlapping window segmentation, which is not tried in the current DCNN method based on spectrogram and pre training. Secondly, for the preprocessing of overlapping window segmentation, we use BLSTM to enhance the time dimension correlation of DCNN speech data, and add attention mechanism to improve the speech segment Feature extraction, which has not been tried by the existing methods combining attention mechanism. Besides, we prove that the pre-trained DCNN model can reduce the influence of small sample to train deep network and improve the accuracy of speech emotion recognition.
2. We demonstrate that the three channels of log Mel-spectrograms (3-D log-Mels) as DCNN input is suitable for affective feature extraction which achieves better performance than LLDs. It is natural and will not lose the important emotional features. Besides, we investigate the effects of different number of channels in Mel-spectrograms.
3. To solve the impact of silent frames and emotion irrelevant frames, an additional attention model is adopted to automatically focus on emotion relevant information. The propose DCNN-BLSTMwA model produce discriminative utterance-level features and the experimental results manifest that this method outperforms the baseline (DNN+ELM) by 16.30% for EMO-DB and 17.26% for IEMOCAP respectively.

The rest of this paper is distributed as follows. Section 2 describes our method and the structure of DCNN-BLSTMwA. The experimental process and the details of the parameter setting are reviewed in section 3. Section 4 analyzes and describes the experimental results. Conclusions are provided in section 5, followed by the future work.

## 2. Proposed Methodology

In this section, we introduce our new method DCNN-BLSTMwA for speech emotion recognition. Firstly, speech samples need to be preprocessed to reduce individual differences. Secondly, we generate the input of DCNNs from the speech signals, the three-channel log Mel-spectrograms (static, deltas, and delta-deltas). And we describe the process of pre-trained and fine-tuning. Then we introduce the structure of DCNN-BLSTMwA which is used to extract emotion features at utterance-level. Finally, we use a three layers fully-connected DNN modal for emotion classification by utterance-level features, see section 2.5 for more details of the DNN.

### 2.1. Preprocessing

The speech emotion database is usually composed of multiple speakers (Neumann and Vu, 2017), whose speech exist differences and variations due to age, gender, cultural etc. Therefore, it is necessary to do speech preprocessing before extracting emotion features. Firstly, zero mean and unit variance are calculated for speech standardization and reducing the impact of individual differences problem. Then, we enhance the speech data according to different speech speed and sampling frequency due to DCNN's training requires a large amount of labeled data and data enhancement can make up for small data samples. Changing the original speed of the speech to a certain extent may change the emotion in the speech, such as speeding it up by 1.5 times or even 2 times. However, we controlled the change of voice speed within the interval of 0.8–1.2, and performed a manual secondary check on the data set after data enhancement, which is equivalent to manually labeling the speech data, proving that this will not change the sample label. Finally, we use data balancing method to make the training data be balanced relatively. Because the number of samples in each class of database is different, there exist the phenomenon of data imbalance influencing the DCNN's training effect. More detail process of data enhancement and balancing datasets will be introduced in section 3.2.

### 2.2. Log Mel-Spectrograms

In recent years, CNN has showed excellent performance in speech emotion recognition (Kim et al., 2017; Weißkirchen et al., 2017). Different from the CRNN model proposed in Trigeorgis et al. (2016), the input of DCNN model is fixed, that is, it should be appropriately calculated from 1-D speech signals. Abdel-Hamid et al. (2014) used the extracted log Mel-spectrograms and organized it into a 2-D array as the CNN input. Chan and Lane (2015) found that 2-D convolution is superior to 1-D convolution in the case of limited data. Motivated by research (Zheng et al., 2018), we use three-channel log Mel-spectrograms as DCNN input. The process of generating three-channel log Mel-spectrograms as follows. Firstly, a pre-emphasis is performed on the speech data to amplify the high frequency part. Then, the hamming window of 25 ms is used to divide it into smaller frames for each speech sentence, the shift is 10 ms. After that, STFT is used to generate the whole log Mel-spectrogram of an utterance. In this paper, we adopt 64 Mel-filter banks from 20 to 8,000 Hz. Then, a context window of 64 frames is adopted to extract the static Mel-spectrogram. The frame shift size of 32 frames is used to generate overlapping segments of Mel spectrogram. The overlapping segments is the key of speech segmentation. As a result, the static Mel-spectrogram is obtained at the size of 64×64. The first 64 represent the number of Mel-filter banks and the other is represent 64 frames of segment window. The length of a segment is 64 frames, that is 655 ms (10 ms × 63 + 25 ms). Some studies have proved that over 250ms can express an emotion enough (Wöllmer et al., 2013), so the segmentation length of this paper is reasonable. In section 3.3, we will compare the relationship between context window size and the recognition accuracy to find the best effects of the segment length. After generating static Mel-spectrogram, the first and second temporal derivatives are employed to obtain other two channers of Mel-spectrograms. We calculated the first and second order regression coefficients along the timeline as the delta and delta-delta coefficients of the Mel-spectrograms. As shown by (1), *m*_*i*_ are log-Mels, and *P*_*i*_ are outputs. $m_{i}^{d}$ are the deltas features of the log-Mels, we use the formula is given by (2). A popular choice for N is 2. And the delta-deltas features $m^{d}d_{i}$ are calculated by taking the time derivative of the deltas, as shown in (3). In this way, three-channel of Mel-spectrograms are extracted. The three-channel of Mel-spectrograms as the DCNN input can be expressed as *X*, *X* ∈ *R*^*F*×*T*×*C*^ where *F* is the number of Mel-filter banks represent the frequency dimension, *T* is the segment length parallel with the frame number in a segment window, and *C* is the number of channels. In this paper, we generate three channers of Mel-spectrogram, so the *C* is 3. The size of *X* is 64 × 64×3, it is similar with a colorful image.

(1) $$\begin{array}{l} m_{i}=\text{log}\left(P_{i}\right) \end{array}$$

(2) $$\begin{array}{l} m_{i}^{d}=\frac{\sum_{n=1}^{N}n(m_{i+n}-m_{i-n})}{2\sum_{n=1}^{N}n^{2}} \end{array}$$

(3) $$\begin{array}{l} m^{d}d_{i}=\frac{\sum_{n=1}^{N}n(m_{i+n}^{d}-m_{i-n}^{d})}{2\sum_{n=1}^{N}n^{2}} \end{array}$$

### 2.3. Pre-training and Finetuning

In this section, we introduce pre-training and finetuning technology. The technology of initialize parameters with pre-trained model is transfer learning which is wildly used in field of image classification (Krizhevsky et al., 2012) and speech recognition (Dahl et al., 2011). Although speech task and image task are two different fields, they get the same network input after preprocessing. The input of DCNN in our model is the spectrum map, which is also a picture. And we proved that the transfer learning is effective through experiments as shown in Table 4. As the number of samples in database is relatively small and with the network become deeper, small-scale samples could easy to cause overfitting. And it can also easy to fall into local solution (Yanai and Kawano, 2015). Firstly, the pre-trained model adopted the initialization weight parameters trained by natural scene image database (ImageNet) of 1,000 classes on the annual competition which is now known as the ImageNet Large Scale Visual Recognition Challenge (ILSVRC) whose recognition accuracy is higher than 95%. Then we finetune the weight by using our speech emotion database. This process can accelerate the speed of network convergence and better fitting the network with small number of samples. More details about fine-tuning of pre-trained DCNN are given in section 3.2.

### 2.4. Architecture of DCNN-BLSTMwA

In this section, we introduce the architecture of DCNN-BLSTMwA model to analyze 3-D log Mel-spectrograms for SER. First, the deep emotion features are extracted from the 3-D log spectrograms by DCNN. Next, we stack the segment-level 3-D DCNN sequence features of a sentence into the utterance-level features. Then we input these utterance-level features into the Bi-directional LSTM (long short-term memory) to extract higher level features with the long-time information. In this way, the high-level features in the two dimensions are obtained. After BLSTM, an attention layer is devoted to highlighting emotion features and reducing the distractions of unrelated segments. Finally, DNN model is adopted to classify the utterance-level features for SER.

#### 2.4.1. DCNN Model

**Figure 2:**
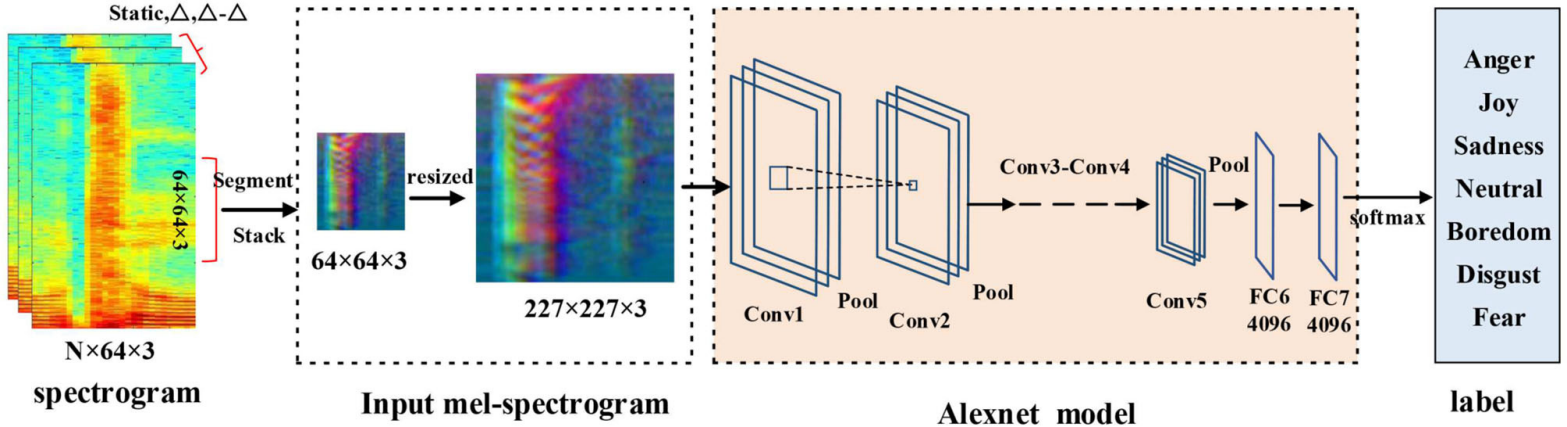
The structure of the DCNN model and the training and fine-tuning process.

As shown in Figure 2, in this paper we use the classic Alexnet network (Abdel-Hamid et al., 2014) as the DCNN model. The DCNN model includes five convolution layers, three max-pooling layers, and two fully connected layers. The size of the convolution kernel of the first layer is 11 × 11 × 96, and the step size is 4 × 4. After the convolution layer of the c1, c2, and c5, there is a max-pooling layer. The pooling size of all pooling layers is 3 × 3, and the step size is 2 × 2. The size of the second convolution kernel is 5 × 5 × 256, while the second and third convolution layer is 3 × 3 × 384. The last convolution kernel of c5 is 3 × 3 × 256. The step size of all convolution layers is 1 × 1. The fully connected layer contains 4,096 linear units, and the output is the segment-level emotional features of the 4,096-dimensional. And the activation function we use Relu. After the last fully connected layer, a dropout layer is followed to minimize the influence of overfitting. Because the input of Alexnet is the fixed size of 227 × 227 pixels, the Mel-spectrograms is 64 × 64 × 3 obtained in section 2.2 this paper. So, we need to reshape the size of DCNN input into 227 × 227 × 3. In this paper, we adopt linear interpolation method to modify the Mel-spectrograms the size, and then input them into DCNN model. More details about parameters setting and fine-tuning of pre-trained DCNN are given in section 3.2.

#### 2.4.2. BLSTM Layer

After extracting and segment-level emotion features with the DCNN model, we stack the segment-level feature sequences into utterance-level with the same length of a sentence. Then we input utterance-level features into BLSTM to extract higher level features for temporal summarization. The structure of combining DCNN and BLSTM can have better performance because DCNN can extract spectral features and BLSTM can extract temporal features from log Mel-spectrograms. These two parts of features are complementary features. Each direction of BLSTM contains 128 cells, after BLSTM we get the 256-dimensional high-level feature representation. We define it as *Y*, *Y* = {*y*_1_, *y*_2_, …, *y*_*i*_, …, *y*_*n*_}, where *y*_*i*_ is a feature representation, *t* is the dimension of BLSTM.

#### 2.4.3. Attention Layer

Attention layer: In a speech, not all the segments are related to emotion such as silent frame and pause segments. These irrelevant features will affect the training and final recognition performance. Attention mechanism can reduce the influence of this problem. Initially, the attention mechanism was applied to image recognition and machine translation. When mimicking human to listen a speech, people often focus on certain strong tones which is more contribute to emotion expression. Therefore, in this paper, attention layer is adopted to focus on emotion features, and weaken the irrelevant ones (e.g., silent frame). Rather than simple operations like max-pooling or average-pooling, attention layer can help to produce the discriminative utterance-level feature representation for final speech emotion classification.

(4) $$\begin{array}{l} \alpha_{i}=\frac{exp(\mu^{T}y_{i})}{\sum_{j=1}^{J}exp(\mu^{T}y_{j})} \end{array}$$

(5) $$\begin{array}{lll} Z & = & \overset{I}{\underset{i=1}{\sum }}\alpha_{i}y_{i} \end{array}$$

**Figure 3:**
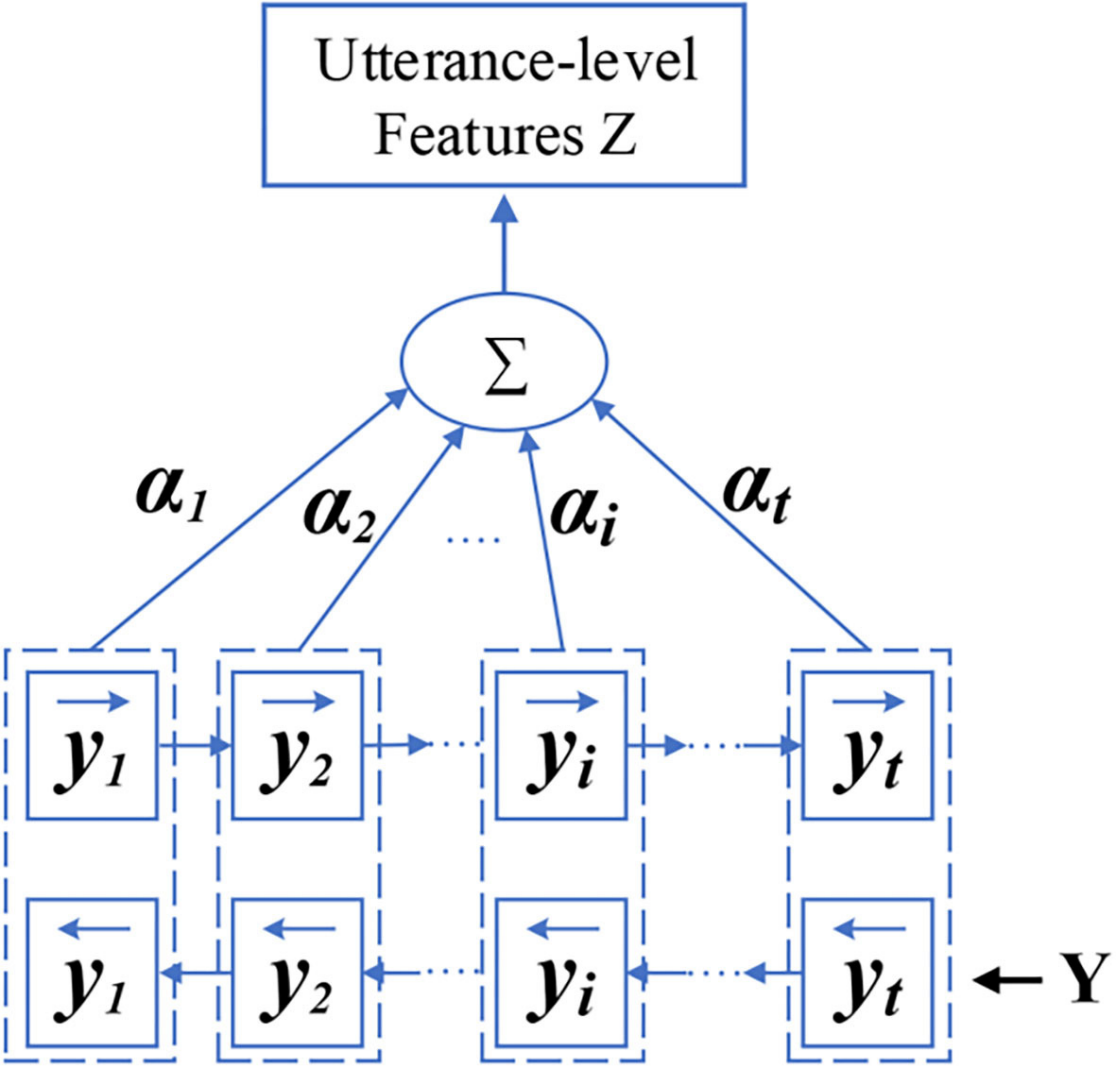
The structure of BLSTM and attention layer and the working process.

As shown in Figure 3, the output of the bidirectional LSTM is *Y*. First, we calculate attention weight α_*i*_. α_*i*_ is obtained from a softmax function as the Equation (4). In evaluate (4), the weight *μ* is obtained by the process of training. Then, we calculate the utterance-level feature representations *Z*, where *Z* is got by performing a weighted sum on *Y*. As shown in Equation (5), we finally produce the higher utterance-level feature *Z* for SER.

### 2.5. DNN Classification

In this section, we introduce the architecture of classification for SER. We gain the final utterance-level features (Z), DNN is used to emotion classification. First, DNN classification model was constructed, and then the utterance-level features were used for DNN training. Finally, we produce the output of DNN as the result of SER. DNN has three layers, the first layer contains 512 cells with a dropout layer. And the second and third layer contains 256 cells, the output is one of the 7 classes emotion. In this way, we achieve the speech emotion recognition by proposed method.

## 3. Experiments

### 3.1. Datasets

In this paper, in order to prove and evaluate the performance of our propose method, we perform abundant speech emotion recognition experiments on Berlin Emotional database (EMO-DB) (Burkhardt et al., 2005) and the Interactive. Emotional Dyadic Motion Capture database (IEMOCAP) (Busso et al., 2008). EMO-DB corpus contains 535 emotional utterance, including seven different emotions: anger, joy, sadness, neutral, boredom, disgust, and fear. The process of this database is that ten professional native German-speaking actors (five men and five women) is asked to imitate the six or seven emotions, and utter 10 sentences in the tone of this emotion. The 10 sentences are five long sentences and five short sentences respectively, which are commonly used in daily communication. The recordings of this database were performed in an extremely quiet room with high-quality equipment at a sampling rate of 16 kHz, 16-bit resolution, and mono channel. The average length of speech file is about 3 s. Twenty participants are required to score the labels, and assess the quality of collected the recordings. IEMOCAP corpus totally contains 10,039 utterances and consists of five sessions, each of which collected the recordings from a pair of actors in scripted and improvised scene (one male and one female). Each utterance is labeled by 3 annotators. If their marks are inconsistent with one another, the data is invalid. The average length of speech file is about 4.5 s at the sample rate of 16 kHz. In this paper, we only use the improvised speech data and use utterances at the emotion labels between four emotion categories, i.e., angry, sad, happy, and neutral. Because the improvised data is more natural and help to the task of SER.

### 3.2. Experiment Setup

**Figure 4:**
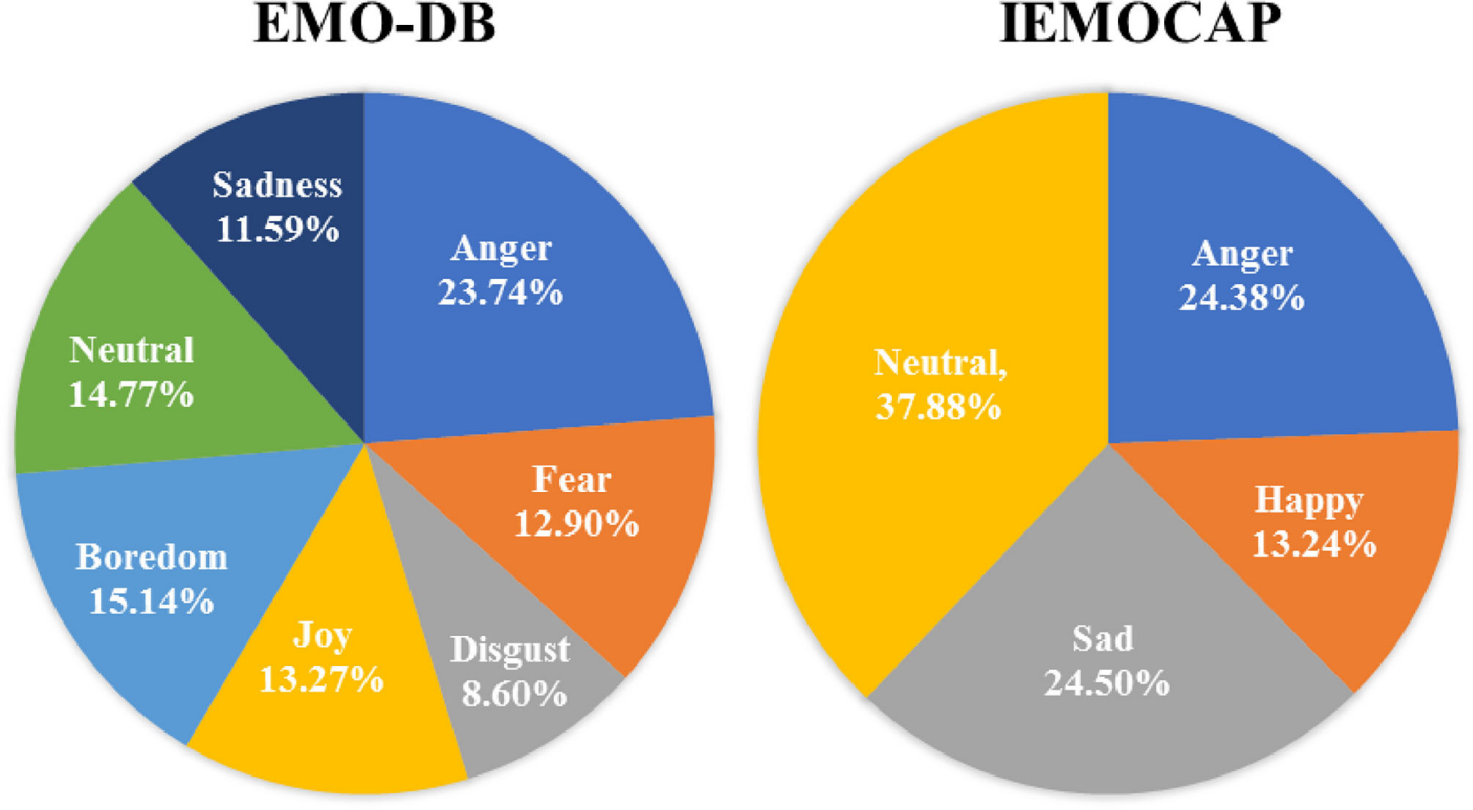
Data distribution of EMO-DB database and IEMOCAP.

There are only 535 speech samples in Berlin emotional database (Burkhardt et al., 2005), and the number of each emotion category is different. And in IEMOCAP database, there are 3,784 speech samples. Although the number of speech samples is enough in IEMOCAP, the number of each emotion category is unbalanced. The problem of the unbalanced data distribution may influence the training effect of DCNN model. The data distribution of EMO-DB and IEMOCAP as shown in Figure 4. It is difficult to train the DCNN model in the case of small amount of data and unbalanced data distribution.

To solve the problem of small samples, we employ the method of data enhancement to expand the speech samples. The details of process: according to the sampling frequency and the playing speed of speech, we conduct data enhancement by adjusting the speed at 0.8, 0.9, 1.0, 1.1, and 1.2 times of the raw speech, respectively. The enhanced data will not lose emotional information, so it can not affect the recognition effect. After data enhancement, we obtain 4 times more than raw speech, and obtain 2,675 sentences at last. To some degree, we solved the problem of small amount of data.

To solve the problem of unbalanced data distribution, we calculate the weight of each category at whole database. The details of process: according to the proportion of each category in all samples, we calculate the weight of the category. Then in the process of DCNN training, network parameters are adjusted according to the weight of each category. For example, the number of Disgust is minimal, it's weight will be largest. And in the process of training, the influence of this class on network parameters will increase accordingly. The smaller the number of categories, the greater the weight and the greater the impact on parameters. Indirectly, the problem of unbalanced data distribution can be eased.

In this way, we reduce the impact of these two issues after data enhancing and balancing weight. Next, we introduce the details of speech segmentation and DCNN training.

The length of each utterance in database is different, in order to better fine-tuning of the DCNN network, we split each speech into equal-length segments. The length of each segment is set as 3 s. If the segment is larger than 3 s, we cut off the redundant part. Otherwise, zeros padding is used for smaller than 3 s. In this paper, we use the PyTorch framework to implement our method. We use the *librosa*−*toolkit* to extract 3-D log Mel-spectrograms (static, delta, and delta-delta). Then we stack the three-channer log Mel-spectrograms, and speech segmentation method is used to obtain segment-level speech data as described in section 2.2. The label of each speech segment is consistent with sample label. Since we normalize the sentences at the equal-length of 3 s, each sentence has the same number of segment-level features, which can be input into DCNN model for training more reasonable.

The training process and details are as follows: First, the network initial parameters are copied from Alexnet training with ImageNet. And then we finetune the DCNN model by using our speech segments, which contain the three-channel log Mel-spectrograms. As shown in Figure 2, the size of spectrogram is 227 × 227 × 3 as DCNN input, and a softmax layer is used to predict emotion categories at training. After finetuning the DCNN model, we take the output of its FC7 layer as segment-level emotional feature Xi. After that, all *X*_*i*_ of an utterance are stacked together to form the utterance-level feature *X*. *X* = {*X*_1_, *X*_2_, …, *X*_*i*_, …, *X*_*n*_}, where *X*_*i*_ is the segment-level features, and *n* is the number of segments, and every utterance has the same *n*. Then we input *X* into BLSTM-Attention model to further extract higher level features. The parameters of the model are optimized by minimizing the cross-entropy objective function. The batch-size is set to 128, the epoch = 20, and the initial learning rate is set to 10^−4^, using Adam optimizer with Nestorov momentum, and the momentum was set to 0.9.

The utterance-level features *X* were input into the BLSTM model to extract the features of temporal information, and the output *Y* is input into an attention layer to highlight emotional feature. Finally, the utterance-level features were classified by the DNN model with three linear layers. The parameters of DNN are also optimized by minimizing the cross-entropy objective function. The batch-size is set to 16, the epoch is 30, and the initial learning rate is set to 5 × 10^−6^, using Adam optimizer with Nestorov momentum, and the momentum was set to 0.9. To get the results more reliable, we performed the “Leave-one-Speaker-out” (LOSO) cross-validation on EMO-DB, eight people are selected as training data, one as validation data, and the last one as test data in each experiment. For IEMOCAP, evaluation is performed in 5-folds, four sessions are selected as training data, one as test data. For each experiment, we test three times and take the average accuracy. Finally, we calculate the unweighted average recall (UAR) of all speakers or sessions as our final experiment results. For each speaker, we test three times and take the average accuracy as the speaker's result. Then, we calculate unweighted average recall (UAR) of all speakers as our final experiment results.

### 3.3. Experiment Results

**Figure 5:**
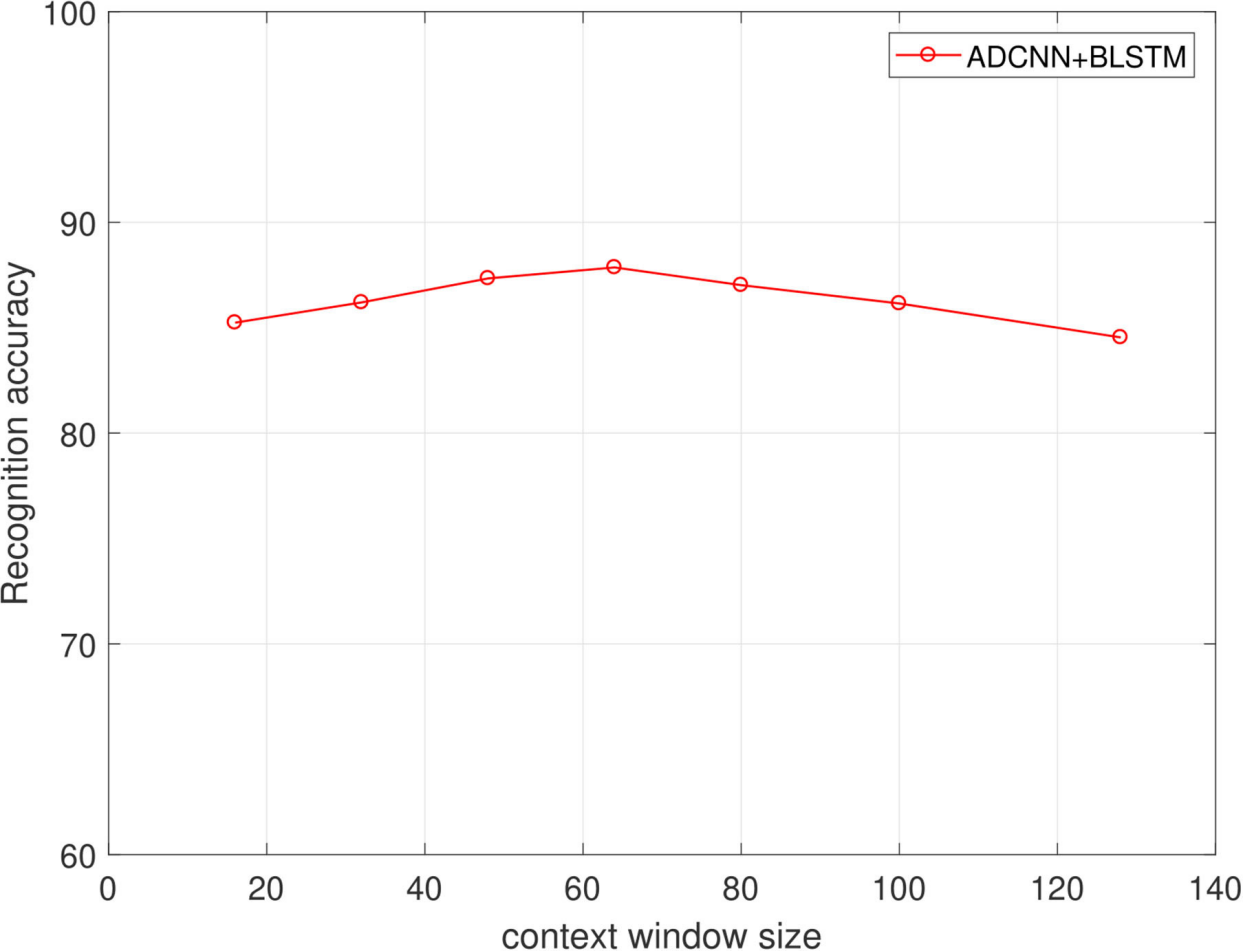
The relationship of context window size and the recognition accuracy(%) on the EMO-DB dataset.

**Figure 6:**
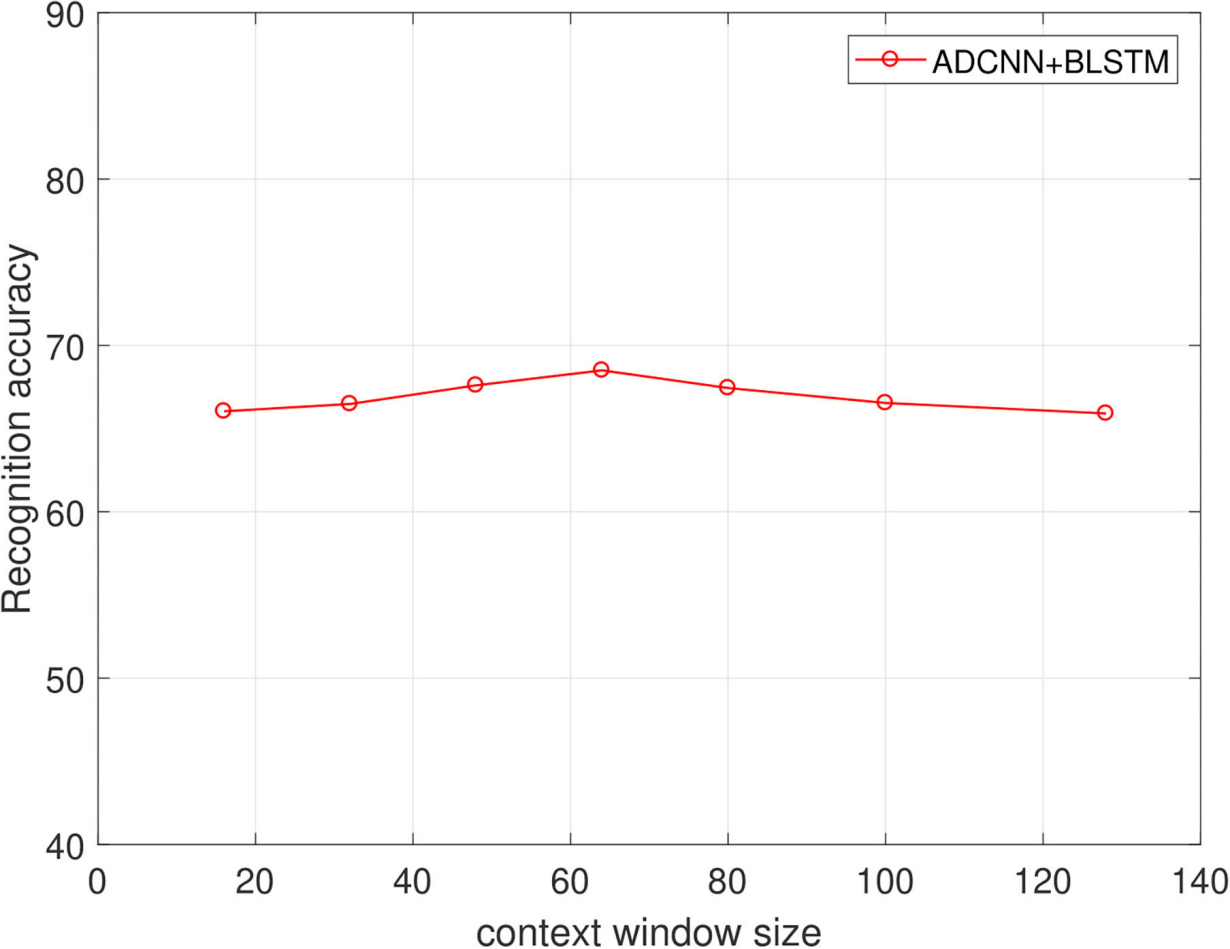
The relationship of context window size and the recognition accuracy(%) on the IEMOCAP dataset.

**Figure 7:**
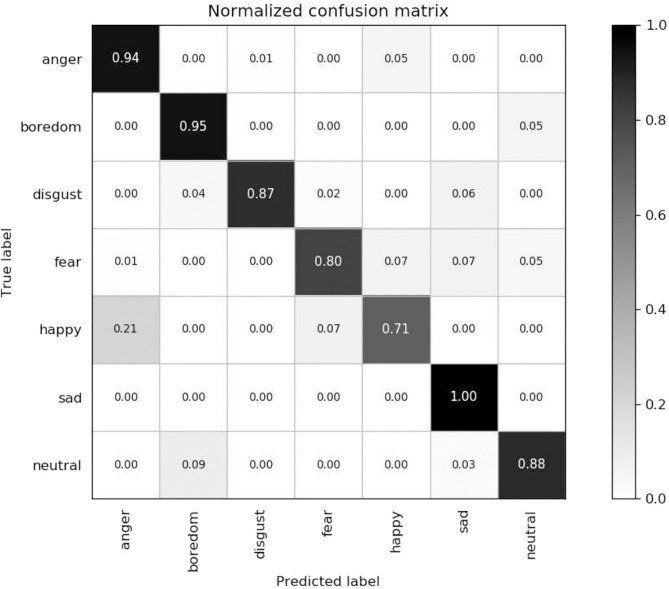
Confusion matrix of ADCNN-BLSTM with an average accuracy of 87.86% on the EMO-DB dataset.

**Figure 8:**
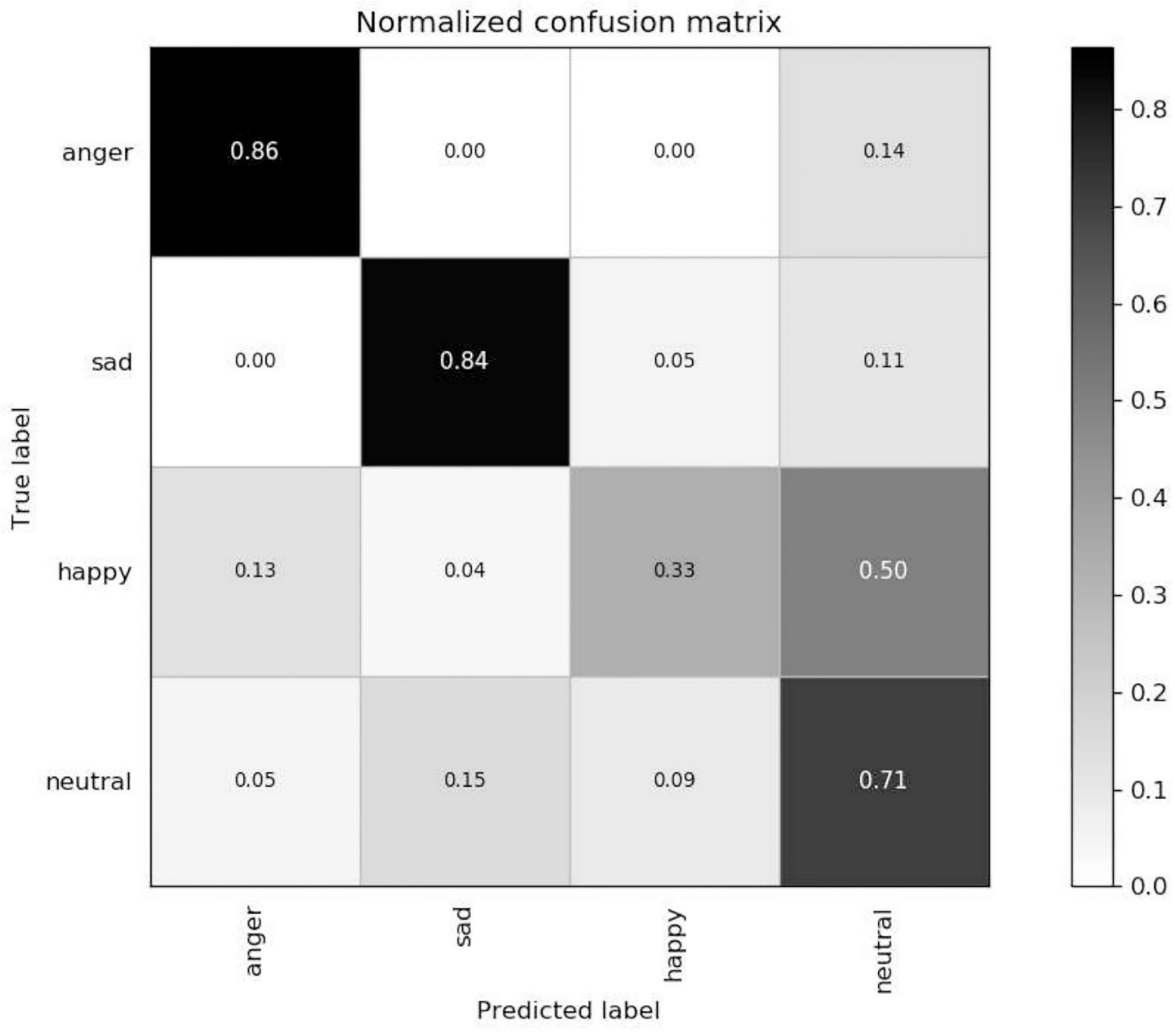
Confusion matrix of ADCNN-BLSTM with an unweighted average recall of 68.50% on the IEMOCAP dataset.

1. Firstly, we tested the effects of the number of channels in log Mel-Spectrograms. We used DCNN-BLSTMwA model to investigate the effects. Specifically, we extracted 3-D log Mel-Spectrograms (static, delta, and delta-delta) and only adopted 1-D Mel-Spectrograms (static) when C = 1, and 2-D Mel-Spectrograms (static and delta) when C = 2 as DCNN input, respectively. The average accuracy as shown in Table 1. When C = 1, average accuracy obtained 80.37% of EMO-DB and 62.38% of IEMOCAP which is better than some traditional methods. This indicates that the high performance of log Mel-Spectrograms. The average accuracy reached 85.05% of C = 2 and 87.86% when C = 3, increasing 4.68 and 7.49%, respectively on EMO-DB. And The average accuracy obtained 66.25% of C = 2 and 68.50% when C = 3, increasing 3.87 and 6.12%, respectively on IEMOCAP. This demonstrated that the first order and second order derivatives of Mel-spectrogram contains helpful emotional information, and the combine of three-channel of Mel-spectrograms can improve the performance for SER.
2. Secondly, we proved the effects of data enhancement by different multiples of samples with different speed of speech as shown in Table 2. Also, we used the proposed model to test it. We found that the accuracy rate increased by 3.80% when we tripled the samples. And it increased by 6.94% when we expanded the data by 5 times. Because we use the different sampling frequency and the playing speed to enhance the samples, the important information will not lose in a speech. The good results prove that data enhancement can help the training of deep network model and improve the final classification accuracy.
3. Table 3 reveals the effects of the attention model. We found that after using the attention model, the average accuracy increased by 7.69% of EMO-DB and 3.36% of IEMOCAP. Abundant experiments proved the powerful performance of attention model, which can focus on important emotional features and help to extract higher utterance-level features and improve the accuracy for SER.
4. Table 4 shows the effects of pre-trained DCNN model. We tested the performance of without pre-trained DCNN model by using the same architecture of our proposed method. The initial parameters were randomly initialized with a standard normal distribution. The average accuracy of without pre-training was 81.31% of EMO-DB and 64.04% of IEMOCAP, we improved 6.55 and 4.46%, respectively by using initial parameters from ImageNet for pre-training. This demonstrates that pre-trained DCNN model not only speeds up the network convergence, but also improves the classification accuracy.
5. Next, in order to find the best effects of the segment length, we designed different segment length to compare the relationship between context window size and the recognition accuracy. we tested context window size ranges in (16, 32, 48, 64, 80, 100, 128), and the effects as shown in Figures 5, 6 of EMO-DB and IEMOCAP database respectively. We found that the context window size of 64 obtained best effects both on these two databases. This demonstrates that the image size of 64 × 64 reshape to 227 × 227 was best size of DCNN's input for training.
6. Then, we compared our proposed approach with several popular methods as shown in Tables 5, 6. We first built the baseline by using 2 convolution layers follow by LSTM. The first convolution layer contains 16 kernels of size 5 × 5 with the stride size of 1 × 1 and the second 32 kernels of size 5 × 5 with the same stride size of 1 × 1. Each convolution layer followed by a Max-pooling layer. The LSTM contains 512 cells with 0.5 dropout rate. Similarly, we also adopted 3-D log Mel-Spectrograms as input to extract the emotional features. The average accuracy of 2 CNN-LSTM is 78.09% of EMO-DB and 58.23% of IEMOCAP which are better than the method of DNN-ELM. As shown in Tables 5, 6, the average accuracy is 87.86% of EMO-DB and 68.50% of IEMOCAP, and our proposed method is better than most of popular SER methods in recent years. This demonstrates the promising performance of our approach.
7. Finally, we present the confusion matrix consistent with the results of DCNN-BLSTMwA to further analyze the recognition accuracy as shown in Figure 7, where the vertical axis represents the true label and the horizontal axis represents the predicted label. In the confusion matrix of EMO-DB, we find that *sad* achieves the highest recognition rate of 1 while *happy* is the lowest accuracy of 0.71, and the *anger* and *boredom* also obtain pretty good recognition rate at 0.94 and 0.95, respectively. Similarly, as shown in Figure 8, the *happy* is also the lowest accuracy of 0.33 on IEMOCAP database. And *anger* and *sad* achieve relatively good result of 0.86 and 0.84, respectively, *neutral* is 0.71. The reason may be that *sad* and *anger* are relatively intense emotions and highly diacritical among these emotions. Thus, these two emotions obtain better results. And to some extent, *happy* emotion features is a little similar with *anger*, so 21% *happy* samples are misclassified into *anger* on EMO-DB. However, there are 50% *happy* samples are misclassified into *neutral* on IEMOCAP. We attribute the misclassification to *neutral* is at the center of these emotions and the *happy* emotion closing to the center is hard to distinguish. In addition, we find that 9% *neutral* are misclassified into *boredom*, it may be the similar activation level between *neutral* and *boredom*. All in all, the average accuracy of 87.86% of EMO-DB and 68.50% of IEMOCAP are promising results and demonstrate the performance of our methods.

**Table 1:** The unweighted average recall (UAR) (%) of the different number of channers (The value of C) in log Mel-Spectrograms.

**The value of C**	**C=1**	**C=2**	**C=3**
EMO-DB	80.37 ± 6.17	85.05 ± 8.75	87.86 ± 6.92
IEMOCAP	62.38 ± 4.58	66.25 ± 6.65	68.50 ± 6.20

**Table 2:** The unweighted average recall (UAR) (%) of different multiples of samples and the effects of data enhancement on EMO-DB database.

**Times (speech speed)**	**1 (1.0)**	**3 (0.9,1.0,1.1)**	**5 (0.8,0.9,1.0,1.1,1.2)**
Average accuracy	80.92 ± 6.38	84.72 ± 7.76	87.86 ± 6.92

*In parentheses is the different speech speed compare with raw samples*.

**Table 3:** The unweighted average recall (UAR)(%) for SER with or without attention model.

**Model (architecture)**	**Without attention (DCNN-BLSTM)**	**With attention (DCNN-BLSTMwA)**
EMO-DB	80.17 ± 6.57	87.86 ± 6.92
IEMOCAP	65.14 ± 4.94	68.50 ± 6.20

**Table 4:** The unweighted average recall (UAR)(%) for SER with or without pre-training.

**Model**	**Without Pre-training**	**With pre-training**
EMO-DB	81.31 ± 4.89	87.86 ± 6.92
IEMOCAP	64.04 ± 5.24	68.50 ± 6.20

**Table 5:** The compare of our proposed approach with several popular methods on EMO-DB.

**Author**	**Method**	**UAR(%)**	**Year**
K. Han	DNN-ELM (Han et al., 2014)	71.56 ± 8.43	2014
Q. Mao	CNN (Mao et al., 2014)	85.20 ± 0.45	2014
S. Zhang	DCNN+DTPM (Zhang et al., 2017)	87.31 ± 6.95	2017
M. Chen	CRNN+Attention (Mingyi et al., 2018)	82.82 ± 4.99	2018
Baseline	2-CNN-LSTM	78.01 ± 6.91	2019
Proposed	DCNN+LSTM+Attention	87.86 ± 6.92	2019

**Table 6:** The compare of our proposed approach with several popular methods on IEMOCAP.

**Author**	**Method**	**UAR(%)**	**Year**
K. Han	DNN-ELM (Han et al., 2014)	51.24 ± 8.24	2014
S. Mirsamadi	RNN+Attention (Mirsamadi et al., 2017)	58.80 ± 4.70	2017
Z. Zhao	Att-BLSTM-FCNs (Zhao et al., 2018)	60.10 ± 4.01	2018
M. Chen	CRNN+Attention (Mingyi et al., 2018)	64.74 ± 5.44	2018
D. Luo	HSF-CRNN (Luo et al., 2018)	63.98 ± 7.56	2018
Baseline	2-CNN-LSTM	58.23 ± 5.21	2019
Proposed	DCNN+LSTM+Attention	68.50 ± 6.20	2019

## 4. Conclusions and Future Work

In this paper, we propose a new method based on pre-trained DCNN model and BLSTM with attention model (DCNN-BLSTMwA) for speech emotion recognition. We first enhanced the speech samples and balanced datasets. Then 3-D log Mel-spectrograms (static, delta, delta and delta) were extracted from the speech signal as DCNN input. DCNN extracted the segment-level features which were stacked to obtain the utterance-level features. Then higher utterance-level features were further extracted through BLSTM with an attention model and finally, the DNN model was used for final SER. Experiments on EMO-DB database have shown the promising performance of our proposed method compared with some popular SER methods. The average accuracy in terms of UAR is 87.86% of EMO-DB and 68.50% of IEMOCAP, respectively, which are better than most popular SER methods of recent years. Additionally, we have also proved the robust performance and feasibility of the method.

In the future, we will try to construct a more stable deep neural network to fit more speech signals for SER. And we will combine the LLDs features and DCNN extracted features to realize speech emotion recognition. In addition, we are going to initial the parameters of other speech emotion database rather than ImageNet. It may help promote the training performance of deep neural model and increase the accuracy for SER.

## Data Availability Statement

Publicly available datasets were analyzed in this study. This data can be found at: http://emodb.bilderbar.info/start.html; https://sail.usc.edu/iemocap/.

## Author Contributions

HZ and RG designed the core methodology of the study, carried out the implement, and drafted the manuscript. YW carried out the experiments and drafted the manuscript. All authors read and approved the final manuscript.

## Conflict of Interest

The authors declare that the research was conducted in the absence of any commercial or financial relationships that could be construed as a potential conflict of interest.
